# Optimization of the composition in a composite material for microelectronics application using the Ising model

**DOI:** 10.1038/s41598-021-81243-2

**Published:** 2021-02-04

**Authors:** Yoshihiko Imanaka, Toshihisa Anazawa, Fumiaki Kumasaka, Hideyuki Jippo

**Affiliations:** 1 Fujitsu Laboratories Ltd., 10-1 Morinosato-Wakamiya, Atsugi, Kanagawa 2430197 Japan; 2 Fujitsu Limited, 4-1-1 Kamikodanaka, Nakahara-ku, Kawasaki, Kanagawa 2110053 Japan

**Keywords:** Engineering, Materials science

## Abstract

Tailored material is necessary in many industrial applications since material properties directly determine the characteristics of components. However, the conventional trial and error approach is costly and time-consuming. Therefore, materials informatics is expected to overcome these drawbacks. Here, we show a new materials informatics approach applying the Ising model for solving discrete combinatorial optimization problems. In this study, the composition of the composite, aimed at developing a heat sink with three necessary properties: high thermal dissipation, attachability to Si, and a low weight, is optimized. We formulate an energy function equation concerning three objective terms with regard to the thermal conductivity, thermal expansion and specific gravity, with the composition variable and two constrained terms with a quadratic unconstrained binary optimization style equivalent to the Ising model and calculated by a simulated annealing algorithm. The composite properties of the composition selected from ten constituents are verified by the empirical mixture rule of the composite. As a result, an optimized composition with high thermal conductivity, thermal expansion close to that of Si, and a low specific gravity is acquired.

## Introduction

Specific new materials are usually adopted in the components and devices of innovative products because the materials make it possible to produce new superior functions. Additionally, in the case where an existing product is modified, the adapting of the material to the characteristic of the component and device is required because the material properties decide and enhance the performance of a product. Therefore, new materials have been developed in earnest in many industrial fields. However, since the conventional Edisonian trial and error approach is costly and time-consuming, it is not possible to meet industry schedules and economic demand. Therefore, the concept of materials informatics uniting material science and engineering with computer and informatic technology is expected to realize rapid and efficient material discovery and development.

As described in the literature^1,2^, materials informatics can be broadly divided into three main parts: data generation (synthesis), data management, and knowledge discovery (analysis). Decades ago, data generation focused on the efficiency of experiments, such as combinatorial materials science and high-throughput ab initio computation based on density functional theory (DFT), was the main topic of interest. Recently, the main topic has shifted to data management and knowledge discovery^3^. Large amounts of collected material data, so-called big data, have been managed and organized in standard global databases to improve the data accessibility by different computational algorithms^4^. Recently, web-based materials informatics platforms have been built using the created materials database^5,6^. Materials simulation, prediction and analysis have been studied using machine learning algorithms such as the decision tree, Bayesian, artificial neural network, perceptron and deep learning. Currently, large-scale calculations are performed using the classical supercomputer, of which the operating speed is determined in accordance with Moor’s law of logic LSI. In the future, when the amount of big data is drastically increased, the classical computer will be unable to manage these large-scale calculations since the scaling of the gate length of the logic LSI will have almost reached the manufacturing limit^7^. To achieve a much higher operating computer speed than that of the classical computer, the realization of quantum computers is expected^8^. There are two types of quantum computers: quantum gate systems and quantum annealing systems. For the quantum gate system, a device featuring a qubit having superposition and entanglement of both the 0 and 1 states has been developed, and the whole computer system has not been commercialized. In contrast, the quantum annealing computer, based on the Hamiltonian framework for quantum mechanics, is currently available^9^. The solution to the optimization problem can be attained by finding the lowest-energy state (ground state) of the Hamiltonian (energy function) in the Ising model using the adiabatic quantum computing approach^10^. In this paper, we introduce a new materials informatics approach in which the optimum composition in a material can be clarified using the Ising model which can be adopted by a quantum annealing system as well as a simulated annealing system.

## Results and discussion

### Composition optimization using Ising machine

Composite materials we examine in this paper are widely used in many applications, from aerospace to biomedical applications and microelectronics^11–13^. The composition of a composite material depends on the application because the requirement of the material varies with the characteristics of the final component product. To date, many studies regarding the empirical mixture rule of composite materials have been performed in metal-based composites as well as ceramic-based and resin-based composite materials^14^. Therefore, once the composition is fixed, the properties of the composite can be predicted by calculating the mixture rule. Also, Fuzzy Preference Selection Method (f-PSI) method and so forth are well-known as the method for selecting the optimal composite material from the given several composite candidates^15^.

However, in the case of inverse problem, the best composition from among many constituent candidates for obtaining the designated properties, with all combinations obtained by changing the composition and constituent, must be calculated until the designed properties are obtained. Thus, since the more the constituent candidates and composition scale are increased, the more the combination is drastically increased, much time is required to calculate all combinations using the classical computer with the von Neumann architecture. For example, in the case of n = 25 cities in the traveling salesman problem, of which the combination is 3.1 × 10^23^ (n!/2n)^16^, it is reported that it takes more than 1.3 billion years to complete all calculations even on a supercomputer, while it is solved in less than 1 min on a machine using heuristics optimization: the Ising model^17^. Therefore, to find the optimum composition of a composite material, we apply the same step in this study. Our approach consists of the following five steps.

- Step 1: Extraction of problems.
- Step 2: Conversion of combinatorial optimization problem.
- Step 3: Formulation of Ising model expression.
- Step 4: Conversion of expression into QUBO.
- Step 5: Computation of QUBO for the optimal solution using Ising machine.

The detailed content of every step is described later.

**Figure 1:**
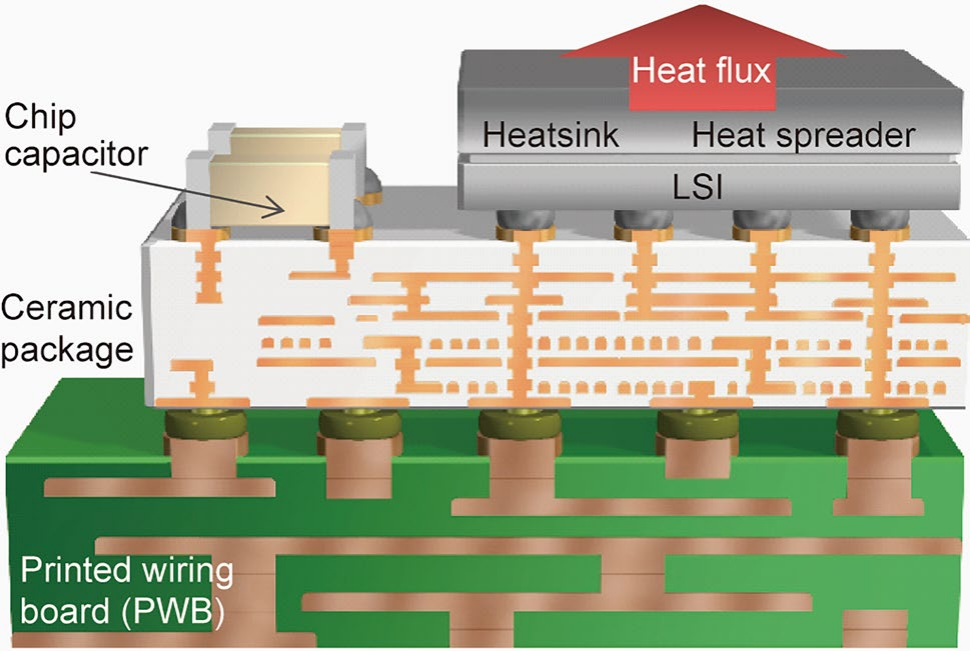
Typical microelectronic package structure of a high-speed computer CPU. Heat generated from the LSI is dissipated from the heat sink and heat spreader attached to the back side of the Si chip.

We choose a heat sink and heat spreader for a high-speed computer as a target component for the materials informatics approach using the Ising model. The heat sink and heat spreader are attached to the backside of the Si chip, establishing a CPU for the high-speed computer^18–20^, as shown in Fig. 1. Under operation, more than 50 W/chip, depending on the computer, is generated on the Si surface^21^. Large amounts of heat should be dissipated from the Si chip efficiently using a heat sink and a heat spreader component to properly operate the circuit on the active side of the Si. Therefore, the following three requirements must be satisfied by the material for a heat sink and heat spreader^22–24^.

1. High thermal conductivity for proper heat dissipation
2. A thermal expansion coefficient close to that of Si for maintaining the attachability with Si
3. A low specific gravity for reducing the mechanical load on the fragile Si and flip-chip bonding structure

To meet the above three requirements simultaneously, we formulate an energy function using the quadratic unconstrained binary optimization (QUBO) format equivalent to the Ising model. The general equation of the Ising model regarding the Hamiltonian H is as follows^25,26^:

$$H = - \mathop \sum \limits_{i \ne j} J_{ij} \sigma_{i} \sigma_{j} - \mathop \sum \limits_{i} h_{i} \sigma_{i} \left( {\sigma = \pm 1} \right)$$

where σ_i_ represents an input variable satisfied as σ ∊ {− 1, + 1}. J_ij_ is a (two-body) interaction given parameter, and h_i_ is referred to as a given magnetic field as a (one-body) parameter. In practice, the model is converted to the equivalent QUBO style with bits of q ∊ {0, 1} instead of σ ∊ {− 1, + 1}. QUBO is obtained only by converting a variable in the Ising model [σ = 2q − 1 or q = (σ + 1)/2].

To adopt the method for solving the discrete optimization problem regarding the composition of a composite material, we introduce variable bit xij ∊ {0, 1} with a material composition (j) of material constituent (i). The values for i (m = 10) and j (n = 100) are applied. Then, m × n = 1000 xij bits are introduced in this solution. All bits are allocated as shown in Table 1.

**Table 1:** A bit pattern of the matrix of x_ij_.

${y}_{j}=0.1\times j$	j/i	1	2	3	**	9	10	${A}_{j}=\sum_{i=1}^{m}{x}_{ij}$
0.1	1	x_1,1_	x_2,1_	x_3,1_	x_**,1_	x_9,1_	x_10,1_	A_1_
0.2	2	x_1,2_	x_2,2_	x_3,2_	x_**,2_	x_9,2_	x_10,2_	A_2_
0.3	3	x_1,3_	x_2,3_	x_3,3_	x_**,3_	x_9,3_	x_10,3_	A_3_
0.4	4	x_1,4_	x_2,4_	x_3,4_	x_**,4_	x_9,4_	x_10,4_	A_4_
0.5	5	x_1,5_	x_2,5_	x_3,5_	x_**,5_	x_9,5_	x_10,5_	A_5_
0.#	#	x_1,#_	x_2,#_	x_3,#_	x_**,#_	x_9,#_	x_10,#_	A_#_
#.#	##	x_1,##_	x_2,##_	x_3,##_	x_**,##_	x_9,##_	x_10,##_	A_##_
9.8	98	x_1,98_	x_2,98_	x_3,98_	x_**,98_	x_9,98_	x_10,98_	A_98_
9.9	99	x_1,99_	x_2,99_	x_3,99_	x_**,99_	x_9,99_	x_10,99_	A_99_
10	100	x_1,100_	x_2,100_	x_3,100_	x_**,100_	x_9,100_	x_10,100_	A_100_
	${F}_{i}=\sum_{j=1}^{n}{x}_{ij}$	F_1_	F_2_	F_3_	F_**_	F_9_	F_10_	

m (value of i): 10, n (value of j): 100.

Ten materials (m = 10) shown in Table 2 are selected as the constituent of the composite in this study.

**Table 2:** Properties of ten materials for the constituent candidate for the simulated annealing of the Ising model in this study.

Properties				
i Constituent candidate	O_i_ Thermal conductivity (W/m K)	P_i_ Thermal expansion (ppm/℃)	Q_i_ Specific gravity (g/cc)	Young’s modulus (GPa)
1. Ag	428	19.6	10.49	82.7
2. Cu	403	16.6	8.94	110
3. Ni	94	13.3	8.9	207
4. C	180	4.5	2.26	10
5. BeO	260	6.4	3.02	330
6. AlN	220	3.5	3.26	270
7. h-BN	600	− 1.5	2.1	10
8. SiC	270	4.3	3.21	400
9. c-BN	850	4.7	3.48	660
10. Diamond	1800	1.1	3.51	1000

Two constrained terms must be introduced to properly map the bit pattern shown in Table 1.

One constraint term F requires that there be at most one bit that is 1 in a certain material constituent column i so that the bit represents a composition of the constituent. This constraint term F is represented by the following formula:

$$\sum_{j=1}^{n}{x}_{ij}={F}_{i}\in \left\{\mathrm{0,1}\right\}$$

$$F=\sum_{i=1}^{m}\left({F}_{i}\left({F}_{i}-1\right)\right)$$

Another constraint term G forces the sum of the compositions of the selected components to be m = 100.

This constraint term G is represented by the following formula:

$$y_{j} = 0.1 \times j\quad A_{j} = \mathop \sum \limits_{i = 1}^{m} x_{ij} \in \left\{ {0,1} \right\}\quad G = \left( {\mathop \sum \limits_{j = 1}^{n} A_{j} y_{j} - 10} \right)^{2}$$

In this study, three objective functions are necessary since three properties are required for the composite material. The first requirement is higher thermal conductivity. The objective function term of thermal conductivity E_TC_ is expressed as follows:

$${E}_{TC}=-{\sum }_{i=1}^{m}{O}_{i}{\sum }_{j=1}^{n}{y}_{j}{x}_{ij}$$

where O_i_ represents the thermal conductivity of constituent i.

The second requirement is a thermal expansion close to that of Si: 3.6 ppm/℃. The objective function term of the thermal expansion E_TE_ is expressed as follows:

$${E}_{TE}={\left(3.6-{\sum }_{i=1}^{m}{P}_{i}{\sum }_{j=1}^{n}{y}_{j}{x}_{ij}\right)}^{2}$$

where P_i_ represents the thermal expansion of constituent i.

The third requirement is a lower specific gravity. The objective function term of the specific gravity E_SG_ is expressed as follows:

$${E}_{SG}={\sum }_{i=1}^{m}{Q}_{i}{\sum }_{j=1}^{n}{y}_{j}{x}_{ij}$$

where Q_i_ represents the specific gravity of constituent i.

The total energy is formulated by adding three objective terms and two constrained terms in the following way:

$${\text{E}} = \alpha {\text{E}}_{{{\text{TC}}}} + \beta {\text{E}}_{{{\text{TE}}}} + \gamma {\text{E}}_{{{\text{SP}}}} + \delta {\text{F}} + \varepsilon {\text{G}}$$

where α, β, γ, δ and ε are hyperparameters that adjust the balance between the constraint terms and the objective functions.

The optimum composition, which satisfies all three requirements simultaneously, is calculated by determining the bit pattern matrix in Table 1 so that the total E is minimized with the simulated annealing process of the Ising model machine^27^. The calculation is performed by one of the Ising machines, i.e., the digital annealer (DA)^28^, for which application-specific CMOS hardware is designed to solve fully connected QUBO problems, using a simulated annealing (SA) algorithm, with a massively parallel architecture to optimize the energy of the Ising model by a Markov chain Monte Carlo (MCMC) search^29,30^. The architecture processes 1024 parallel bits that are fully connectable through 16-bit weights.

As a result of the simulated annealing process with the Ising machine, the following bits are chosen as the optimized answer: x_2, 38_, x_5, 3_, x_6, 2_, x_7, 9_, x_9, 19_, x_10, 29_. One of the optimum compositions in this study is 38Cu-3BeO-2AlN-9h-BN-19c-BN-29Diamond (percent by volume), depending on the hyperparameters α, β, γ, δ and ε. This is the final answer to the inverse problem solved by the Ising model machine. The role of hyperparameter is determining the function weight of every term in total energy equation and normalizing the energy value of every term. In this case, we set α: 0.001, β: 0.01, γ: 0.01, δ: 80 and ε: 100, respectively. The total calculation time of simulated annealing process was less than 10 s, like the 25 cities traveling salesman problem described above^16^.

### Verification of composite material properties using mixture rule

We verify the properties of the optimized composition of a composite material using the mixture rule^31^. The mixture rules for the thermal conductivity κ of composites, normally applied to composite materials, are shown below. The first equation is the mixing rule where the heat flux direction and the constituent materials are arranged in parallel, and the second equation is where the heat flux direction and constituent materials are arranged perpendicularly. The isotropic composites of the type in which constituent particles are distributed in a matrix match the empirical logarithmic law well

$$\kappa = \mathop \sum \limits_{i = 1}^{m} V_{i} \kappa_{i} \quad {\raise0.7ex\hbox{$1$} \!\mathord{\left/ {\vphantom {1 \kappa }}\right.\kern-\nulldelimiterspace} \!\lower0.7ex\hbox{$\kappa $}} = \mathop \sum \limits_{i = 1}^{m} {\raise0.7ex\hbox{${V_{i} }$} \!\mathord{\left/ {\vphantom {{V_{i} } {\kappa_{i} }}}\right.\kern-\nulldelimiterspace} \!\lower0.7ex\hbox{${\kappa_{i} }$}}\quad log\;\kappa = \mathop \sum \limits_{i = 1}^{m} V_{i} \;log\;V\kappa_{i}$$

κ_i_ is the thermal conductivity of constituent material i, V_i_ is the volume fraction of constituent material i.

In the mixture rules for thermal expansion coefficient α of the isotropic composites, the following Turner equation is well known

$$\alpha = {{\mathop \sum \limits_{i = 1}^{m} \alpha_{i} V_{i} K_{i} } \mathord{\left/ {\vphantom {{\mathop \sum \limits_{i = 1}^{m} \alpha_{i} V_{i} K_{i} } {\mathop \sum \limits_{i = 1}^{m} V_{i} K_{i} }}} \right. \kern-\nulldelimiterspace} {\mathop \sum \limits_{i = 1}^{m} V_{i} K_{i} }}$$

α_i_ is the thermal expansion coefficient of constituent material i, V_i_ is the volume fraction of constituent material i, K_i_ is the Young’s modulus of constituent material i.

The specific gravity ρ of a composite material can be calculated by the following equation if the secondary phases are not formed between constituent materials in a composite:

$$\rho = \sum_{i=1}^{m}{V}_{i}{\rho }_{i}$$

ρ_i_ is the specific gravity of constituent material i, V_i_ is the volume fraction of constituent material i.

**Figure 2:**
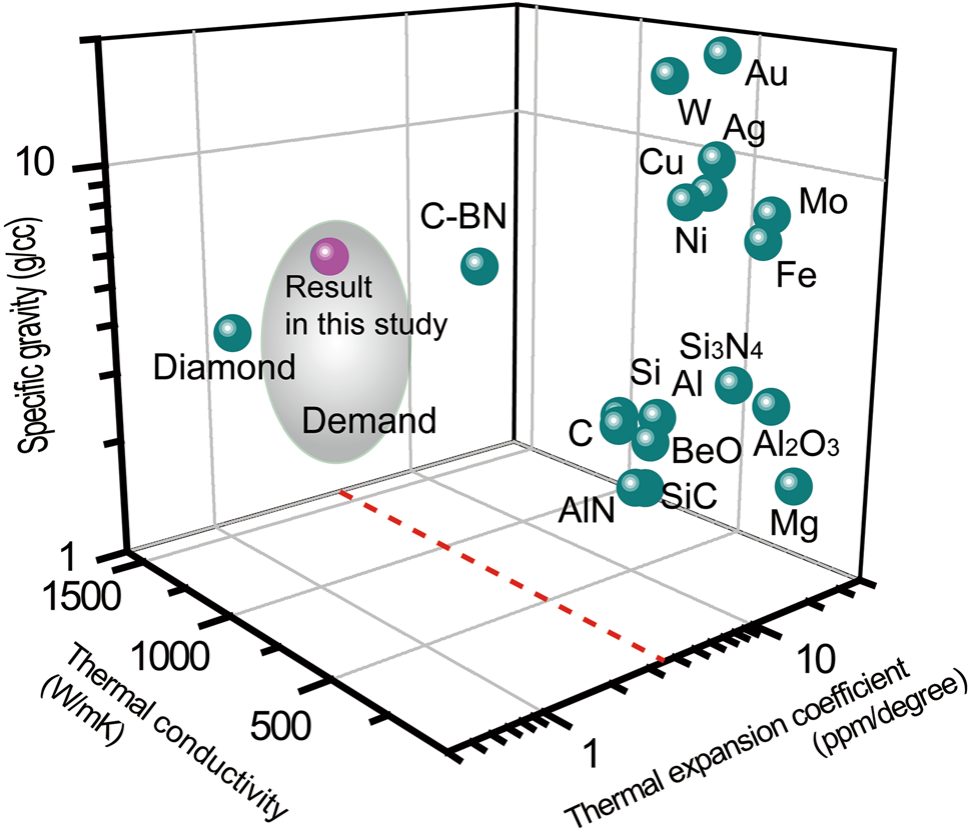
Calculated three required properties of the composite material optimized using the Ising model compared with the existing material candidate for the heat sink. The composite material simultaneously satisfies all three demands: high thermal conductivity, thermal expansion close to that of Si, and a low specific gravity.

Table 3 shows the calculated properties of the optimized composite material obtained by the mixture rule. The composite material with its composition optimized by the Ising model satisfies all three demanded properties, as shown in Fig. 2.

**Table 3:** Calculated properties of the optimized composite material obtained by the Ising model using the mixture rule.

Properties			
Composite material	Thermal conductivity (W/m K) calculated by logarithmic law	Thermal expansion (ppm/℃) calculated by Turner equation	Specific gravity (g/cc) calculated by simple mixture rule
38Cu-3BeO-2AlN-9h-BN-19c-BN-29Diamond	724	3.56	5.42

The composite material, which includes diamond, will be able tobe produced by conventional^32,33^ and modern 3D-printing material processing^34,35^.

As described above, the target of this study is the material for the heat sink and heat spreader of a high-speed computer driven by a silicon active chip. Recently, similar types of heat sinks and heat spreaders have been needed for power electronics applications. For example, the power amplifier of the base station of the mobile terminal for 5G and post 5G generates much higher heat than that of a high-speed computer^36^. In that application, by adopting the thermal expansion of a material of the active device, i.e., SiC or GaN, the same energy function of the Ising model can be utilized.

Furthermore, other additional requirements such as the cost of material, eco-friendliness, and so forth, can be covered by adding the designated objective function term, though only three objective functions regarding thermal conductivity, thermal expansion and specific gravity are specified in this study. Moreover, this composition optimization approach is used for the dielectric composite material in the same manner, since the dielectric constant can be verified using the mixture rule as follows.

The first equation is the parallel mixing rule, where the composite constituent material is aligned parallel to the electric field, and the second equation is the series rule, where the composite constituent material is aligned in series with the electric field. The isotropic composite constituent material is aligned randomly and empirically obeys the logarithmic law^37^

$$\varepsilon = \mathop \sum \limits_{i = 1}^{m} V_{i} \varepsilon_{i} \quad {\raise0.7ex\hbox{$1$} \!\mathord{\left/ {\vphantom {1 {\varepsilon }}}\right.\kern-\nulldelimiterspace} \!\lower0.7ex\hbox{${\varepsilon }$}} = \mathop \sum \limits_{i = 1}^{m} {\raise0.7ex\hbox{${V_{i} }$} \!\mathord{\left/ {\vphantom {{V_{i} } {\varepsilon_{i} }}}\right.\kern-\nulldelimiterspace} \!\lower0.7ex\hbox{${\varepsilon_{i} }$}}\quad ln\;\varepsilon = \mathop \sum \limits_{i = 1}^{m} V_{i} \;ln\;\varepsilon_{i}$$

ε is the dielectric constant of the composite, ε_i_ is the dielectric constant of constituent material i, V_i_ is the volume fraction of constituent material i.

## Conclusions

We demonstrated that a new materials informatics approach that incorporates the Ising model is valuable for the optimization of the composition in a material. At this time, we use the Ising machine with fully connected 1024 variables in size. Since the variables are enhanced to 8192 by improving pairing application-specific CMOS hardware recently, much larger-scale materials informatic problem can be solved. Also, this approach can be applied to the quantum annealing machine because the QUBO format we studied is compatible with that machine.

This new materials informatics approach will become a more powerful tool as the development of quantum annealers and modified Ising machine enable more massive and faster calculations.

## Methods

In this paper, no materials were used since a theorical approach was discussed. The methodology used was described throughout the paper.

## Data Availability

Authors can confirm that all relevant data are included in the paper.
